# Characterization of Two *Trichinella spiralis* Adult-Specific DNase II and Their Capacity to Induce Protective Immunity

**DOI:** 10.3389/fmicb.2018.02504

**Published:** 2018-11-05

**Authors:** Xin Qi, Xin Yue, Yue Han, Peng Jiang, Fan Yang, Jun J. Lei, Ruo D. Liu, Xi Zhang, Zhong Q. Wang, Jing Cui

**Affiliations:** Department of Parasitology, Medical College, Zhengzhou University, Zhengzhou, China

**Keywords:** trichinellosis, *Trichinella spiralis*, DNase II, larval invasion, protective immunity

## Abstract

Deoxyribonuclease II (DNase II) is a widespread endonuclease, which can degrade the DNA. *Trichinella spiralis* adult-specific DNase II-1 (TsDNase II-1) and DNase II-7 (TsDNase II-7) were identified in excretory–secretory (ES) or surface proteins of adult worm (AW) and intestinal infective larvae (IIL) using immunoproteomics with early infection sera. The aim of this study was to characterize the two *T. spiralis* DNase II enzymes and to investigate their role as potential vaccine candidate target molecules. The cDNA sequences of the two DNase II enzymes from 3 days old AWs of *T. spiralis* were cloned and expressed. The sequencing results showed that the complete cDNA sequences of the two DNase II enzymes were 1221 and 1161 bp long, and the predicted open reading frames encoded 347 and 348 amino acids, respectively. On Western blot analysis, natural TsDNase II-1 and TsDNase II-7 in the crude extracts of IIL, AWs, and newborn larvae (NBL) and AW ES proteins were recognized by both anti-rTsDNase II-1 and anti-rTsDNase II-7 sera. Indirect immunofluorescence test and qPCR showed that the two DNase II enzymes were highly expressed at AW and NBL stages and were mainly located at the cuticle and stichosome of the nematode. Vaccination with the two recombinant DNase II enzymes triggered prominent humoral responses that exhibited significant immune protection against *T. spiralis* larval infection, as demonstrated by the notable reduction in intestinal AW and muscle larva burdens. Specific antibodies to the two molecules evidently inhibited the *in vitro* parasite invasion of enterocytes and participated in the killing of NBL by an antibody-dependent cell-mediated cytotoxicity (ADCC) mode. The enzymes DNase II-1 and DNase II-7 are the potential target molecules for anti-*Trichinella* vaccine for blocking both larval invasion and development.

## Introduction

Trichinellosis is a major foodborne parasitosis with a cosmopolitan distribution because it was ranked as the seventh major foodborne parasitic disease all over the world (Food and Agriculture Organization of the United Nations [FAO]/World Health Organization [WHO], 2014). *Trichinella* infection is caused by the ingestion of raw or improperly cooked meat of pigs and other animals infected with *Trichinella* larvae. Human *Trichinella* infection is principally caused by *Trichinella spiralis*, and pork and pork products are the dominant infectious source (Cui and Wang, 2011; Murrell and Pozio, 2011). China is one of the countries with high prevalence of porcine *Trichinella* infection and high number of trichinellosis patients (Cui et al., 2013a; Jiang et al., 2016; Bai et al., 2017). From 2004 to 2009, 1387 cases of human trichinellosis in 15 outbreaks had been reported, and four deaths were caused by this disease (Cui et al., 2011). Trichinellosis also has a great hazard on human health, meat productions, and food safety. Therefore, development of a vaccine is needed to interrupt the *Trichinella* transmission in domestic animals and from animals to humans (Zhang et al., 2018).

After digestion by the enzymes in the stomach, the encapsulated muscle larvae (ML) of *T. spiralis* are liberated from the contaminated meat, which then migrate to the intestine and develop into intestinal infective larvae (IIL) (Ren et al., 2011). The IIL penetrate the intestinal columnar epithelium and grow into sexually mature adult worms (AWs) after undergoing four molts. The AWs live in the intramulticellular niche of the columnar epithelium of the intestine mucosa. After copulating, female AWs produce the newborn larvae (NBL), which are distributed throughout the body by lymphatic and blood circulation, then the NBL penetrate and encapsulate the skeletal muscles of new hosts to complete their life cycle (Despommier, 1998). The AW is an important stage of the *T. spiralis* lifecycle. If the development of the IIL to AW is interrupted or the AWs are promoted to be expelled from the host’s intestine, the NBL production would be blocked, which will prevent trichinellosis.

Deoxyribonuclease II (DNase II) belongs to a unique family of endonucleases, which can degrade the DNA to produce 3′-phosphorylated and 5′-hydroxyl products. The function of these enzymes exhibits an acid pH optimum and does not require divalent cations or cofactors for efficient catalysis (Evans and Aguilera, 2003). Previous studies on DNase II homologs showed that DNase II has an important effect in the development and homeostasis of *C. elegans* and *Drosophila* (Lyon et al., 2000; Mukae et al., 2002). Compared with enzymes from other organisms, *T. spiralis* has an extensive expansion of this secreted DNase II-like protein family with about 125 genes in its genome (Mitreva et al., 2011). Twenty-six *T. spiralis* genes, which putatively encode DNase II homologs, have been identified from the AWs and NBL cDNA library, 15 of these genes are expressed in NBL and 11 genes are expressed in AWs (Liu et al., 2008; Liao et al., 2014). In our previous studies, *T. spiralis* adult-specific DNase II-1 (TsDNase II-1, GenBank: AAY32316.1)/TsDNase II-7 (GenBank: AAY32322.1) was identified from AWs and IIL excretory–secretory (ES) or surface proteins by immunoproteomics (Liu R.D. et al., 2015, 2016a,b; Wang et al., 2017).

In this study, we cloned and expressed the two *T. spiralis* adult-specific DNase II (TsDNase II-1 and TsDNase II-7) enzymes. Their biological characteristics were investigated, and their role as potential vaccine candidate target molecules against trichinellosis was evaluated in mice.

## Materials and Methods

### Ethics Statement

This study was conducted in accordance with the National Guidelines for Experimental Animal Welfare (Minister of Science and Technology, China, 2006). The animal experimental protocols were approved by the Life Science Ethics Committee, Zhengzhou University (No. SCXK 2015–0005).

### Scheme of This Study

**FIGURE 1:**
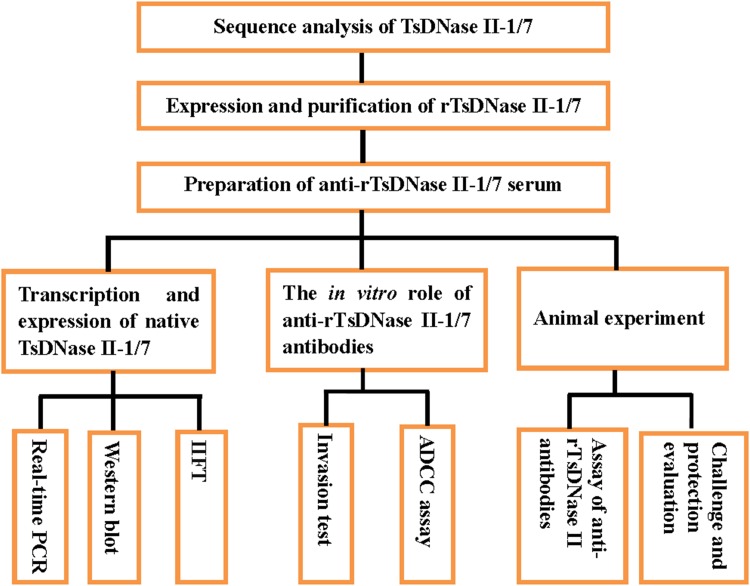
Scheme of characterization of TsDNase II and their capacity to induce protective immunity.

Characterization of TsDNase II and their capacity to induce protective immunity was carried out based on the scheme (Figure 1).

### Parasite, Animals, and Cells

The *T. spiralis* isolate (ISS534) was maintained in mice in our laboratory, which was originally obtained from an infected domestic pig in Henan Province, China. Female BALB/c mice, which were 6 weeks old, were purchased from the Experimental Animal Center of Henan Province. Intestinal epithelial cells (IECs) were prepared from normal fetal mouse intestines, and they were susceptible to the invasion by *T. spiralis* larvae (Ren et al., 2011; Long et al., 2015).

### Collection of Various Stage Worms and Protein Preparation

The mice experimentally infected with 300 *T. spiralis* ML were killed at 42 days post-infection (dpi). The carcasses were minced and digested at 43°C for 4 h with 0.33% pepsin (1:31000; Sigma) and 1% HCl. After filtration and sedimentation, the ML were counted by stereomicroscopy (Li et al., 2010). The IIL was collected from small intestines at 6 hours post-infection (hpi) (Liu R.D. et al., 2015). Sixty mice were orally infected with 5000 ML. Thirty mice were sacrificed at 3 dpi, and the rest were sacrificed at 6 dpi. The small intestine was cut along its length, washed by phosphate buffered saline (PBS), cut into pieces, and incubated in PBS at 37°C for 1.5 h on a 300 μm sieve. The AWs were released from the intestinal debris and collected by filtration with a 200 μm sieve and were subjected to differential sedimentation for 30 min. After washing using PBS supplemented with 100 U penicillin/ml and 100 μg streptomycin/ml, the worms were centrifuged at 600 ×*g* for 10 min, collected, and counted by microscopy (Sun et al., 2015). The NBL were collected from 6 days old female AWs, which were cultivated for 24 h in RPMI-1640 medium with 10% fetal bovine serum (FBS) (Long et al., 2015; Wu et al., 2016).

Crude extracts of worms of various stages (ML, IIL, 3 and 6 days old AWs, NBL) and 3 days old AW ES proteins were prepared (Wang et al., 2011; Li et al., 2015). Briefly, different worms were suspended in deionized water. The worms underwent five cycles of freezing-thawing. The worms were homogenized on ice in a glass tissue grinder, and then the worm fragments were further homogenized with ultra-sonication (200 W, 3 s, 99 times, 0°C). The supernatant was centrifuged at 15,000 g for 20 min at 4°C and was collected.

### Sequence Analysis

The complete cDNA sequence of TsDNase II-1 (GenBank: AY963695.1) and TsDNase II-7 (GenBank: AY963701.1) were analyzed by the open reading frame (ORF) finder program in the NCBI website. The conserved domains of the two proteins were predicted using the Conserved Domain Database of NCBI (Long et al., 2014). Amino acid sequences of TsDNase II-1 and TsDNase II-7 were submitted to an online tool^1^ for predicting their molecular weights (MWs) and isoelectric points (pI). The signal peptide was predicted using the SignalP 4.1 Server (Petersen et al., 2011). The amino acid sequences were aligned with DNase II of other *Trichinella* species and other organisms using Clustal X, and phylogeny was estimated using the neighbor-joining (NJ) method by MEGA 6.0 (Liu L.N. et al., 2013; Zhang et al., 2015).

### Real-Time Quantitative PCR (qPCR) Analysis

Total RNA of *T. spiralis* worms of different stages (ML, IIL, 3 and 6 days old AWs, and NBL) was extracted by Trizol (Invitrogen) and then transcribed into first-strand cDNA with the PrimeScript RT Reagent Kit (Takara) (Ren et al., 2013). The transcription levels of TsDNase II-1 and TsDNase II-7 genes were quantified by qPCR (Liu R.D. et al., 2013). Specific primers for qPCR of TsDNase II-1 (5′-TCTGCAACAGTACCCGCTTTC-3′; 5′-GGCGCAGTCAAGTTCTTATGCTA-3′) and TsDNase II-7 (5′-GAGAAAATCATGCGCTGGTC-3′; 5′-ATTACATGCACAGTAAA CAGTAG-3′) were designed by Primer Premier 5. The transcription levels of TsDNase II-1 and TsDNase II-7 genes were standardized by the transcription level of the housekeeping gene glyceraldehyde-3-phosphate dehydrogenase (GAPDH) (5′-AGATGCTCCTATGTTGGTTA TGGG-3′; 5′-GTCTTTTGGGTTGCCGTTGTAG-3′) and then determined by the comparative Ct (2^-ΔΔCt^) method (Schmittgen and Livak, 2008; Sun et al., 2018a). Each experiment was repeated three times, and triplicates of each sample were performed.

### Expression and Identification of TsDNase II-1 and TsDNase II-7

Total RNA was extracted from 3 days old AWs using Trizol (Invitrogen). The complete cDNA sequences of TsDNase II-1 and TsDNase II-7 were amplified by PCR with the specific primers carrying BamHI and HindIII restriction enzyme sites **(in bold**) (TsDNase II-1: 5′-CA**GGATCC**GATTACCAATGCAAAGAACA-3′ and 5′-CC**AAGCTT**TTAGGTGCATCCA TCCAAGT-3′; TsDNase II-7: 5′-CG**GGATCC**GACTTCCAATGTTTCCAAGA-3′ and 5′-CCC**AAGCTT**TTAGGTGCATGCATCCAAGT-3′). The PCR products were cloned into the vector pMD19-T (Takara, China) and then subcloned into the vector pQE-80L (Novagen, United States) with an N-terminal His_6_ tag. The recombinant plasmids were transformed into the *E. coli* DH5α strain (Sangon Biotech, China) for expression. The Ni-NTA-Sefinose resin (Sangon Biotech, China) was used to purify rTsDNase II-1 and rTsDNase II-7. After purification, rTsDNase II-1 and rTsDNase II-7 were identified by sodium dodecyl sulfate- polyacrylamide gel electrophoresis (SDS-PAGE) analysis (Liu et al., 2017).

### Preparation of Immune Serum

Twenty-two mice were injected subcutaneously with 20 μg rTsDNase II-1 or rTsDNase II-7 emulsified with complete Freund’s adjuvant. Two boost immunizations were carried out at an interval of 10 days using the same amount of rTsDNase II-1/7 emulsified with incomplete Freund’s adjuvant (Cui et al., 2013c; Song et al., 2018a). The immunized mice were bled at 10 days after the final immunization, and the serum samples were isolated. Pre-immune serum and *T. spiralis* infection serum were collected from mice before infection and at day 42 post-infection and were used as controls for the *in vitro* invasion test and *in vitro* antibody-dependent cell-mediated cytotoxicity (ADCC) experiment.

### Western Blot Analysis

Samples consisted of crude extracts of ML, IIL, 3 and 6 days old AW, NBL, and AW ES proteins, rTsDNase II-1, and rTsDNase II-7; these proteins were analyzed by SDS-PAGE with 10% polyacrylamide gels (Wang L. et al., 2013). The gel was subsequently transferred onto nitrocellulose membranes (Millipore, United States) (Wang et al., 2012). The membrane was blocked at 37°C for 1 h with 5% skim milk in tris buffered saline with Tween 20 (TBST) (pH 7.4) and then incubated overnight at 4°C with 1:100 dilutions of anti-rTsDNase II-1 serum or anti-rTsDNaseII-7 serum. Anti-mouse IgG-horse radish peroxidase (HRP) conjugate (Southern Biotechnology, United States) was diluted to 1:10,000 and incubated with the membranes at 37°C for 1 h. The substrate was 3, 3′-diaminobenzidine tetrahydrochloride (DAB; Sigma-Aldrich) (Wang B. et al., 2013; Yang et al., 2015).

### Indirect Immunofluorescence Test (IIFT)

Anti-rTsDNase II-1 serum and anti-rTsDNase II-7 serum were utilized to detect the expression and localization of natural TsDNase II-1 and TsDNase II-7 in the *T. spiralis* worms of various stages (Zhang et al., 2013; Sun et al., 2018b). The worms were fixed using 4% paraformaldehyde, then embedded in paraffin after dehydration, and microtomed into 3 μm-thickness sections. The indirect immunofluorescence test (IIFT) with intact worms and their sections was performed (Liu et al., 2018). The worms and sections were blocked with 5% goat serum for 1 h and then incubated with 1:10 dilution of different sera (anti-rTsDNase II-1 or anti-rTsDNase II-7 serum, *T. spiralis* infection serum, or pre-immune serum). Subsequently, they were reacted for 1 h at 37°C with anti-mouse IgG-FITC conjugate (1:100; Santa Cruz Biotechnology, United States) and finally observed under fluorescent microscopy (Olympus, Tokyo, Japan) (Cui et al., 2015b).

### The *in vitro* Invasion Test

To observe the suppression effects of anti-rTsDNase II-1 and anti-rTsDNase II-7 sera on the invasion of intestinal epithelial cells (IECs) by *T. spiralis*, the IIL were utilized for the invasion test (Yang et al., 2015). The IECs were grown to confluence on 6-well culture plates. One hundred and fifty IIL were firstly mixed with 2 ml DMEM semisolid medium with 1.75% agarose, and then the mixture containing larvae was added onto the IEC monolayer (Xu et al., 2018). Before use, the medium was supplemented with 1:50 to 1:800 dilutions of anti-rTsDNase II-1 or anti-rTsDNase II-7 sera and 1:50 dilutions of pre-immune serum or *T. spiralis*-infected mouse serum (ManWarren et al., 1997). After being incubated for 2 h at 37°C, the invaded worms within the monolayer were examined. The invaded and migrated larvae within the monolayer were counted as invaded larvae, whereas the larvae suspended in media were numbered as non-invaded larvae (Cui et al., 2015a; Zhang et al., 2016). Four independent tests for anti-rTsDNase II-1 or anti-rTsDNase II-7 sera, infection serum, and pre-immune serum were performed, and three repeats were served to evaluate larva invasion rate for each kind of serum (Long et al., 2015).

### The *in vitro* ADCC Experiment

The cytotoxicity of anti-rTsDNase II-1 and anti-rTsDNase II-7 antibodies on the NBL were evaluated as reported before (Moskwa, 1999; Liu L.N. et al., 2015). One hundred and fifty NBL were cocultured for 72 h at 37°C with 1 × 10^5^ mouse peritoneal exudate cells (PECs) in a 96-well plate in the presence of various sera (1:5 to 1:1,000 dilutions of anti-rTsDNase II-1 serum, anti-rTsDNase II-7 serum, pre-immune serum, or *T. spiralis*-infected serum). The larva viability was assessed according to its mobility and morphology under light microscopy. The live NBL were mobile and wriggling, and the dead NBL were straight and immobile or disintegrated (Jiang et al., 2012). The cytotoxicity was calculated as the percentage of dead NBL to total number of NBL in each experiment (Liu et al., 2018).

### Immunization of Mice and Specific Antibody Determination

One hundred mice were divided into five groups of 20 animals each. Vaccination groups of mice were inoculated subcutaneously with 20 μg proteins (rTsDNase II-1, rTsDNase II-7, or 1:1 mixed of these two proteins). Equal volume of Freund’s adjuvant was used to emulsify the rTsDNase II protein, and the mice were boosted twice at an interval of 10 days (Gu et al., 2017). Two control groups received only adjuvant or PBS. Tail blood was collected on 0, 10, 20, and 30 days after the first vaccination (Liu et al., 2017).

Indirect enzyme-linked immunosorbent assay (ELISA) with rTsDNase II-1, rTsDNase II-7, their mixtures, or AW ES proteins was used to detect serum specific total IgG and its subclass (IgG1 and IgG2a) in vaccinated mice (Long et al., 2014; Pompa-Mera et al., 2014). Plate was coated with 1 μg/ml of rTsDNase II or 2.5 μg/ml AW ES proteins at 4°C overnight and then blocked with 5% nonfat milk in PBST. After washing, the plate was incubated with immune serum (1:100) at 37°C for 1 h. After washing again, HRP-conjugated anti-mouse IgG, IgG1, or IgG2a (1:5,000; Sigma-Aldrich) was added and incubated for another 1 h. The compound *o*-phenylenediamine dihydrochloride (OPD; Sigma-Aldrich) was used as the substrate for HRP. The optical density (OD) at 490 nm was determined by an ELISA reader (Tecan, Schweiz, AG, Switzerland).

### Challenge Experiment

To evaluate immune protection, each mouse was orally administrated with 300 ML at 10 days following the third immunization. Intestinal AWs were collected from 10 mice at 5 dpi (Yang et al., 2010), and the ML at 42 dpi were recovered by artificial digestion of the carcasses of the other 10 mice (Liu et al., 2015a). Immune protection was evaluated according to the number of AWs or ML recovered from the vaccinated group compared with those from the PBS group (Liu et al., 2015b; Xu et al., 2017).

### Statistical Analysis

The statistical analyses of the data were performed using SPSS for Windows, version 17.0. Pearson correlation analysis was used for analyzing the relationship between serum dilutions and the inhibition of IIL invasion or ADCC. The analyses of intragroup or intergroup differences of transcription levels in the different stages of *T. spiralis*, cytotoxicity, specific antibody responses, and immune protection were performed using one-way ANOVA or Student’s *t*-test. For parasite invasion of IECs assay, the analyses of intergroup differences were performed by chi-square test. All data were expressed as the mean ± standard deviation (SD), and the differences were considered significant at *P* < 0.05.

## Results

### Sequence Analysis of the cDNA Encoding the TsDNase II-1 and TsDNase II-7 Proteins

**FIGURE 2:**
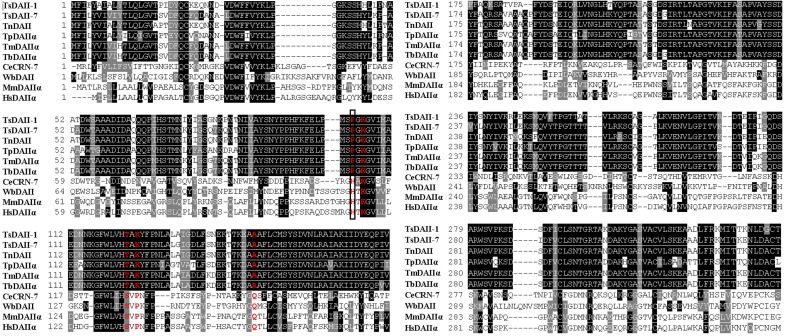
Amino acid sequence alignment of *Trichinella spiralis* TsDNase II-1 (AAY32316.1) and TsDNase II -7 (AAY32322.1) with the DNase II of other *Trichinella* species and other organisms. The other DNase II were WbDAII (*Wuchereria bancrofti*, EJW85434.1), CeCRN-7 (*Caenorhabditis elegans*, NP_498817.1), TnDAII (*Trichinella nativa*, OUC49849.1), MmDAIIα (*Mus musculus*, NP_034192.1), HsDAIIα (*Homo sapiens*, NP_001366.1), TpDAIIα (*Trichinella pseudospiralis*, KRY93508.1), TmDAIIα (*Trichinella murrelli*, KRX36402.1), and TbDAIIα (*Trichinella britovi*, KRY55822.1). The putative active sites are marked in red, and the predicted catalytic site is boxed.

**FIGURE 3:**
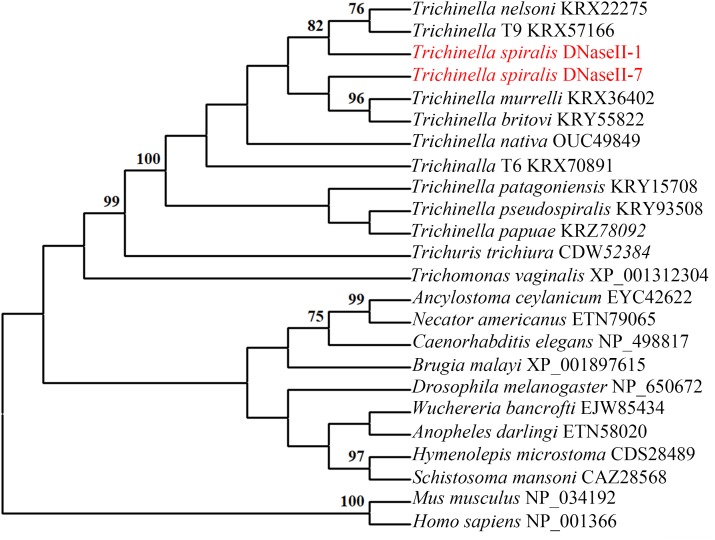
Phylogenetic tree of TsDNase II-1, TsDNase II-7, and DNase II from other *Trichinella* species and other organisms using the neighbor-joining method. The trees were rooted using *Homo sapiens* and *Mus musculus*.

The full-length of the TsDNase II-1 cDNA sequence was 1221 bp, and the predicted ORF (1–1044 bp) encoded 347 amino acids. The predicted MW of TsDNase II-1 is 38.06 kDa with a pI of 8.85. The complete TsDNase II-7 cDNA sequence was 1161 bp long, and the predicted ORF (4–1050 bp) encoded 348 amino acids. The predicted MW of TsDNase II-7 is 38.22 kDa, and its pI is 7.56. In addition, TsDNase II-1 and TsDNase II-7 have signal peptides, and both the cleavage sites are positioned between 18 and 19 amino acids. The putative active sites and the predicted catalytic sites of TsDNase II-1 and TsDNase II-7 are shown in Figure 2. The phylogenetic analysis of TsDNase II-1 and TsDNase II-7 with the DNase II of other organisms shows that TsDNase II-1 has the closest evolutionary status with *T. nelsoni* and *Trichinella* genotype T9, while TsDNase II-7 is genetically related to *Trichinella murrelli* and *Trichinella britovi* (Figure 3).

### Transcription Levels of TsDNase II-1 and TsDNase II-7 in the Different Stages of *T. spiralis*

**FIGURE 4:**
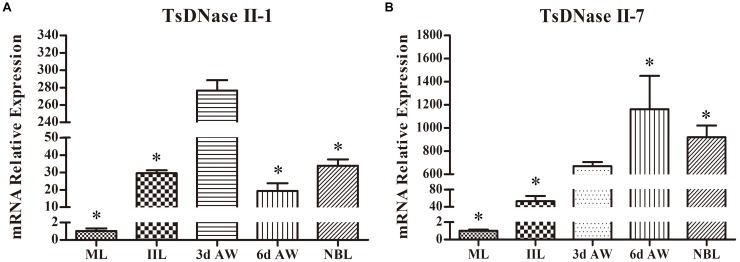
qPCR quantification of TsDNase II-1 and TsDNase II-7 mRNA at the various stages of *T. spiralis* (ML, IIL, 3 and 6 days old AW, and NBL). **(A)** Transcription level of TsDNase II-1 in 3 days old AW was statistically significantly higher than those of the worms in other stages (ML, IIL, 6 days old AW, and NBL) (*P* < 0.05). **(B)** Transcription levels of TsDNase II-7 in 3 days old AW was obviously higher than those of the ML and IIL stages but lower than those of the 6 days old AW and NBL stages (*P* < 0.05). ^∗^*P* < 0.05 compared with 3 days AW.

A qPCR analysis was performed to quantify the transcription of the TsDNase II-1 and TsDNase II-7 genes in the *T. spiralis* worms of various stages (ML, IIL, 3 days old AW, 6 days old AW, and NBL), and the results showed that the transcription of TsDNase II-1 and TsDNase II-7 was detected at all the developmental stages of *T. spiralis*. The TsDNase II-1 transcription level in 3 days old AWs was statistically significantly higher than those of the worms in the other stages (ML, IIL, 6 days old AW, and NBL) (*P* < 0.05) (Figure 4A). The TsDNase II-7 transcription level in 3 days old AWs was obviously higher than those of the ML and IIL stages but lower than those of the 6 days old AW and NBL stages (*P* < 0.05) (Figure 4B).

### Cloning and Expression of TsDNase II-1 and TsDNase II-7

**FIGURE 5:**
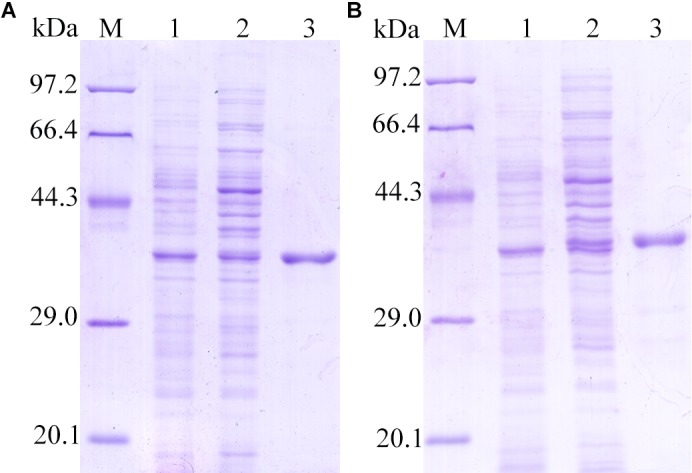
SDS-PAGE analysis of rTsDNase II-1 **(A)** and rTsDNase II-7 **(B).** M: protein marker; 1: the lysates of bacteria carrying recombinant plasmid before induction; 2: the lysates of the induced bacteria carrying recombinant plasmid; 3: purified rTsDNase II-1 **(A)** and rTsDNase II-7 **(B)**.

The cloning and sequencing results revealed that the cDNA ORF of TsDNase II-1 and TsDNase II-7 without signal peptides were about 987 bp (57–1044 bp) and 990 bp long (61–1050 bp), and the predicted MW were 36.01 kDa and 36.17 kDa, respectively. The bacteria harboring pQE-80L/TsDNase II-1 or pQE-80/TsDNase II-7 were induced for 6 h at 37°C with 1 mM isopropyl β-D-1-thiogalactopyranoside (IPTG). On SDS-PAGE analysis, it was observed that the rTsDNase II-1 and rTsDNase II-7 proteins have the consistent MW with the predicted combined size (Figure 5).

### Western Blotting Identification of rTsDNase II-1 and rTsDNase II-7

**FIGURE 6:**
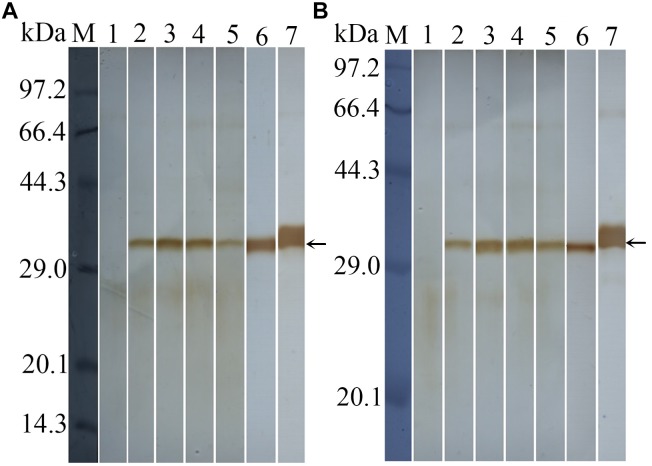
Western blotting of natural TsDNase II-1 **(A)** and TsDNase II-7 **(B)** in the crude extracts from the different stages of *T. spiralis* and 3 days old AW ES proteins. Natural TsDNase II-1 or TsDNase II-7 proteins in the crude extracts from the different stages of *T. spiralis* and 3 days old AW ES proteins were identified using anti-rTsDNase II-1 **(A)** or anti-rTsDNase II-7 **(B)** serum. Lane 1: ML; lane 2: IIL; lane 3: 3 days AW; lane 4: 6 days old AW; lane 5: NBL; lane 6: 3 days old AW ES proteins; lane 7: rTsDNase II-1 **(A)** and rTsDNase II-7 **(B)**.

Western blot analysis revealed that rTsDNase II-1 and rTsDNase II-7 were recognized by anti-rTsDNase II-1 and anti-rTsDNase II-7 sera, respectively. Both natural TsDNase II-1 and TsDNase II-7 proteins in the crude extracts of the different stages of *T. spiralis* other than ML and 3 days old AW ES proteins were identified using the anti-rTsDNase II-1 serum or the anti-rTsDNase II-7 serum (Figure 6), indicating that TsDNase II-1 and TsDNase II-7 were expressed in the different stages of *T. spiralis* but not in the ML stage.

### Expression and Immunolocalization of TsDNase II-1 and TsDNase II-7 at Various Stage Worms

**FIGURE 7:**
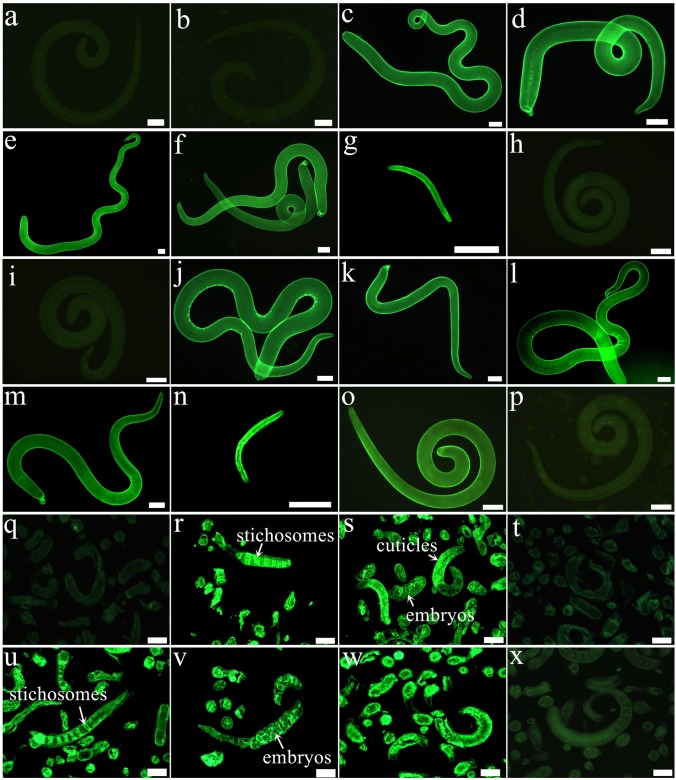
Expression and immunolocalization of TsDNase II-1 and TsDNase II-7 at the various stages of *T. spiralis*. **(a–g)** IIFT with fresh whole worms detected by anti-rTsDNase II-1 serum. There is distinct fluorescence staining on the cuticle external surface of 3 days old female **(c)** and male adults **(d)**, 6 days old female **(e)** and male adults **(f)**, and NBL **(g)** but not on the cuticle of ML **(a)** and IIL **(b)**. **(h–n)** IIFT with intact worms using anti-rTsDNase II-7 serum. Apparent staining is seen on the epicuticle of 3 days old female **(j)** and male **(k)** adults, 6 days old female **(l)** and male adults **(m)**, and NBL **(n)** but not found on the epicuticle of ML **(h)** and IIL **(i)**. The ML probed using infection serum **(o)** or pre-immune serum **(p)** were used as a positive or negative control. **(q–s)** IIFT with worm sections probed by anti-rTsDNase II-1 serum. Staining was seen in IIL **(r)** and 3 days old AW **(s)** but not in ML **(q)**. **(t–v)** IIFT with worm sections incubated with anti-rTsDNase II-7 serum. Immunostaining is observed in IIL **(u)** and 3 days old AW **(v)** but not in ML **(t)**. The ML sections incubated with infection serum **(w)** or pre-immune serum **(x)** were utilized as a positive or negative control. Scale bars: 50 μm.

Results of IIFT with intact worms using anti-rTsDNase II-1 and anti-rTsDNase II-7 sera revealed that green fluorescence staining was homogenously distributed along the external surface of the 3 and 6 days old AWs and NBL, whereas no staining was detected on the cuticles of the ML and IIL stages (Figure 7). Moreover, immunostaining was observed at early, midterm, and late embryo stages (Supplementary Figure 1). After worm sections were incubated using anti-rTsDNase II-1 or anti-rTsDNase II-7 sera, immunostaining was located at the epicuticles and embryos of the 3 days old AWs and stichosome of IIL but not on the ML tissue sections.

### Suppression of Parasite Invasion of IECs by TsDNase II Specific Antibodies

**FIGURE 8:**
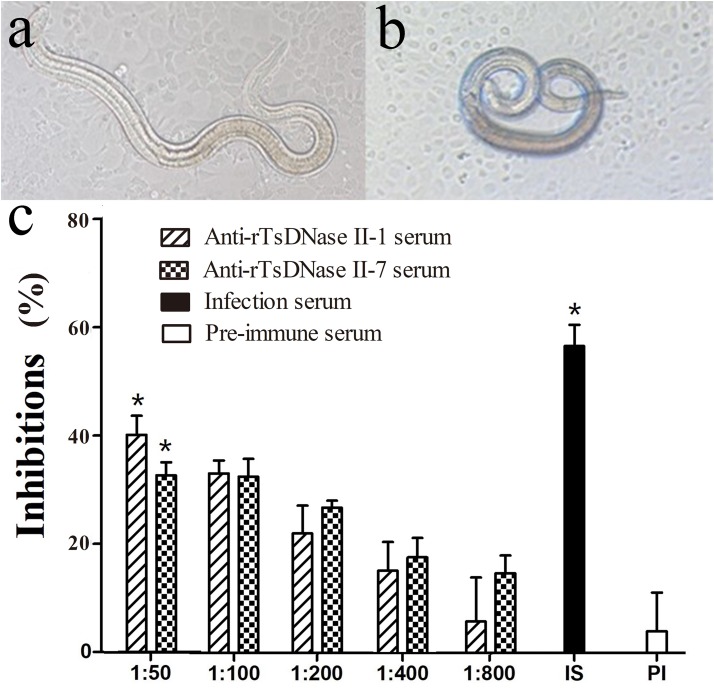
Suppression of parasite invasion of enterocytes by different dilutions of anti-rTsDNase II-1 and anti-rTsDNase II-7 sera. The invaded larvae within monolayer **(A)** and the non-invaded larvae suspended in media **(B)** were observed and counted after incubating with the IEC monolayer for 2 h. **(C)** The percentage of inhibition was standardized by PBS control and was shown as the mean ± SD of three independent experiments. The statistical difference (*P* < 0.05) was indicated with an asterisk (^∗^) relative to pre-immune serum.

After incubating with the IEC monolayer for 2 h, the invaded larvae and non-invaded larvae were observed and numbered (Figures 8A,B). When 1:50 dilutions of different sera were used, the larval invasion rate in the monolayer supplemented with anti-rTsDNase II-1 serum, anti-rTsDNase II-7 serum, infection serum, pre-immune serum, and PBS were 51.97, 58.82%, 37.50%, 83.02%, and 87.42%, respectively (Supplementary Table 1). The inhibition rate of pre-immune serum, anti-rTsDNase II-1 serum, anti-rTsDNase II-7 serum, and infection serum were 4.01%, 40.13%, 32.65%, and 56.69%, respectively. The inhibition of immune serum and *T. spiralis* infection serum were distinctly greater than those of pre-immune serum ($\chi_{1}^{2}$ = 105.012, $\chi_{2}^{2}$ = 70.118, *P* < 0.0001). The inhibition effects of immune sera had a correlation with dilutions of anti-rTsDNase II-1 serum (*r* = -0.881, *P* < 0.0001) and anti-rTsDNase II-7 serum (*r* = -0.891, *P* < 0.0001) and exhibited a decreasing trend with a serum dilution increase (*F*_1_ = 20.749, *F*_2_ = 27.655, *P* < 0.0001). Nevertheless, we failed to observe the evident inhibition of pre-immune serum on larva invasion (Figure 8C).

### The *in vitro* ADCC Assay

**FIGURE 9:**
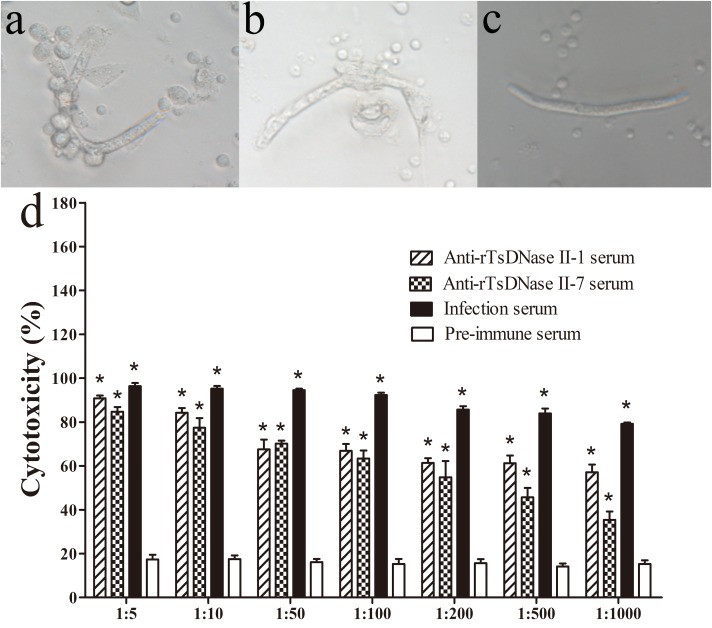
The *in vitro* ADCC assays against *T. spiralis* newborn larvae. Mouse peritoneal exudates cells (PECs) adherence to NBL **(A)**, dead NBL **(B),** and live NBL without PECs adherence **(C)** were observed after ADCC assay. The cytotoxicity has a correlation with dilutions of anti-rTsDNase II-1 and anti-rTsDNase II-7 sera **(D)**. Asterisks (^∗^) indicate that cytotoxicity of anti-rTsDNase II-1 serum, anti-rTsDNase II-7 serum, and infection serum was significantly greater than that of pre-immune serum (*P* < 0.01).

The ADCC assay revealed that after incubation at 37°C for 72 h, anti-rTsDNase II-1 and anti-rTsDNase II-7 sera mediated the adhering of the PECs to the NBL and the subsequent killing of the NBL (Figure 9). When 1:100 dilutions of anti-rTsDNase II-1 and anti-rTsDNase II-7 sera were used, the ADCC resulted in the remarkable death of NBL (66.84% and 63.39% cytotoxicity), in comparison with the NBL treated by pre-immune serum (15.30%, *t*_1_ = 26.420, *t*_2_ = 22.599, *P* < 0.0001). The cytotoxicity had a correlation with the dilutions of anti-rTsDNase II-1 (*r* = -0.927, *P* < 0.0001) and anti-rTsDNase II-7 sera (*r* = -0.771, *P* < 0.0001). The cytotoxicity had a decreasing trend following the increase of serum dilutions (*F*_1_ = 69.569, *F*_2_ = 68.231, *P* < 0.0001).

### Specific Antibody Responses Triggered by Vaccination With rTsDNase II

**FIGURE 10:**
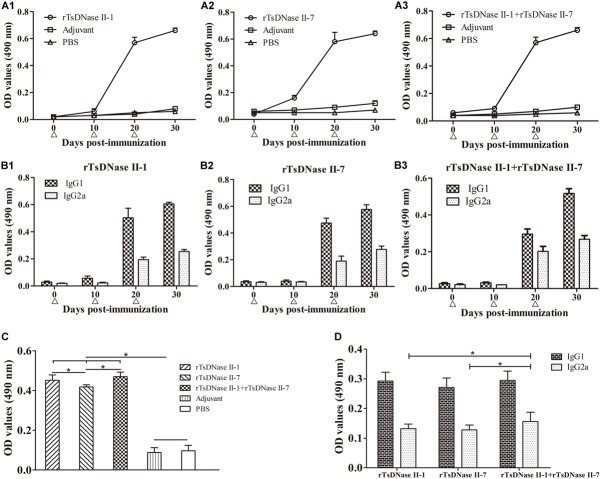
Antibody responses in mice immunized with rTsDNase II-1, rTsDNase II-7, or rTsDNase II-1 + rTsDNase II-7. **(A)** Levels of specific total IgG against rTsDNase II-1 **(A1)**, rTsDNase II-7 **(A2)** and rTsDNase II-1 + rTsDNase II-7 **(A3)** in the serum of immunized mice. **(B)** Levels of specific IgG subclass (IgG1 and IgG2a) against rTsDNase II-1 **(B1)**, rTsDNase II-7 **(B2),** and rTsDNase II-1 + rTsDNase II-7 **(B3)**. Specific total IgG **(A)** and its subclass IgG1 and IgG2a **(B)** were assayed by ELISA with rTsDNase II-1, rTsDNase II-7, or their mixtures. **(C)** Specific total IgG in five groups of immunized mice after the third vaccination was determined by ELISA with AW ES proteins. **(D)** Specific IgG subclass (IgG1 and IgG2a) in the three groups of immunized mice after the third vaccination was determined by ELISA with AW ES proteins. OD values are the mean ± SD of the specific antibody level of 10 mice in each group. Vaccination time point is signed by a triangle (Δ). ^∗^*P* < 0.05.

Specific total IgG and its subclass (IgG1 and IgG2a) antibodies in serum from mice vaccinated with rTsDNase II-1, rTsDNase II-7, or their mixtures were measured by ELISA at different time points after first vaccination. Total IgG and its subclass IgG1 and IgG2a of anti-rTsDNase II-1 and anti-rTsDNase II-7 antibodies and the antibodies against the mixture of these two proteins increased obviously after the second vaccination and reached the highest level following the third vaccination (Figure 10), but no obvious detectable specific antibodies were identified in mice inoculated with only adjuvant or PBS. Mice vaccinated with rTsDNase II-1 exhibited higher levels of IgG1 than IgG2a at 20 and 30 days after vaccination (*t*_20d_ = 13.318, *t*_30d_ = 56.608, *P* < 0.0001), which was similar to the levels of IgG1 and IgG2a in mice vaccinated with rTsDNase II-7 (*t*_20d_ = 17,507, *t*_30d_ = 22.466, *P* < 0.0001) or rTsDNase II-1 + rTsDNase II-7 (*t*_20d_ = 7.821, *t*_30d_ = 24.942, *P* < 0.0001), indicating that the predominant IgG subclass triggered with the two rTsDNase II proteins was IgG1; however, the IgG2a response was also elicited after the second vaccination, and the concurrent Th1/Th2 immune response was elicited by immunization with rTsDNase II.

When specific antibodies of vaccinated mice were assayed by ELISA using AW ES proteins, the total IgG level was not significantly different between mice vaccinated with rTsDNase II-1 and rTsDNase II-1 + rTsDNase II-7 (*P* < 0.01), but total IgG level of the above-mentioned two groups of vaccinated mice was higher than those of mice vaccinated with rTsDNase II-7 (*P* > 0.05). The IgG1 level was not statistically different among the three groups of vaccinated mice (*F* = 1.889, *P* > 0.05); the IgG2a level also was not significantly different between the two groups of mice vaccinated with rTsDNase II-1 or rTsDNase II-7 (*t* = 0.564, *P* > 0.05), but the IgG2a level of mice vaccinated with rTsDNase II-1 + rTsDNase II-7 was higher than those of mice vaccinated with rTsDNase II-1 (*t* = 2.171, *P* < 0.05) and rTsDNase II-7 (*t* = 2.510, *P* < 0.05).

### Immune Protection Against Challenge With *T. spiralis* ML

The protection was investigated in mice immunized with rTsDNase II-1, rTsDNase II-7, or the mixture of these two proteins after the larval challenge infection. The results demonstrated that vaccination of mice with rTsDNase II-1, rTsDNase II-7, or the mixture of these two proteins produced a 40.36%, 34.86%, and 49.54% reduction in AWs at 5 dpi (*F* = 4.028, *P <* 0.05), in comparison with the PBS group. The AW burden of the three groups of immunized mice was significantly lower than those of the mice that received only adjuvant (*t*_1_ = 3.593, *t*_2_ = 2.922, *t*_3_ = 5.513, *P <* 0.01). The reduction of muscle larva burden in the three groups of vaccinated mice at 42 dpi was 50.43%, 42.33%, and 57.79%, separately (*F* = 1.408, *P* > 0.05), compared with the PBS group (Table 1). The muscle larva burden of the three groups of immunized mice was obviously lower than those of the adjuvant group (*t*_1_ = 6.812, *t*_2_ = 5.363, *t*_3_ = 6.815, *P <* 0.001).

**Table 1:** Intestinal adult worms (AWs) and muscle larvae per gram (LPG) recovered from vaccinated mice after being challenged by 300*T. spiralis* larvae.

Groups	Intestinal adult worms		Muscle larvae per gram (LPG)	
	No. of adults recovered	Reduction (%)	No. of larvae recovered	Reduction (%)
rTsDNase II-1^∗^	65 ± 16	40.36	2020 ± 645	50.43
rTsDNase II-7^∗^	71 ± 14	34.86	2357 ± 751	42.33
rTsDNase II-1 + 7^∗^	55 ± 9	49.54	1720 ± 822	57.79
Adjuvant	95 ± 21	12.84	3889 ± 581	4.56
PBS	109 ± 23	-	4075 ± 832	-

*^∗^The adult worm and muscle larva burden of vaccinated mice was significantly lower than those of the adjuvant group (*P* < 0.01).*

## Discussion

The DNase enzymes perform their biochemical functions by degrading DNA through the hydrolysis of its phosphodiester backbone, and they have been found to exert an important role in pathogen invasion in evading host defense. For instance, the extracellular Group A *Streptococcus* DNase is a virulent factor for disease progression; the DNase can help the bacteria to escape the killing by the reticular structural extracellular traps (ETs) formed by the DNA and proteases released by the host’s macrophages and neutrophils in the process of pathogen infection (Buchanan et al., 2006; Yousefi et al., 2008). The expression of TatD-like DNase was involved in *Plasmodium falciparum* virulence, and it was indispensable for parasite growth in the host; a reduction in parasitemia and delayed death was seen in mice vaccinated with recombinant TatD-like DNase from *P. berghei* or *P. chabaudi* after challenge (Chang et al., 2016). An endonuclease homologous to DNase II located at the teguments of the *Schistosoma japonicum* female adult may play a key role in parasite-host interaction (Hou et al., 2015). When *Brugia malayi* microfilariae were incubated with human polymorphonuclear leukocytes (PMNs), the PMNs released ETs that capture the microfilariae. However, after the DNase was added into the medium, the DNase eliminated the PMN attachment to microfilariae (McCoy et al., 2017). The enzyme DNase II has been detected in the ES and surface proteins of *T. spiralis* AW, IIL, and ML stages, and it affects the host immune system (Cui et al., 2013b; Wang et al., 2014, 2017; Liu et al., 2016a,b). Hence, DNase II may have a major function in parasite-host interaction during *T. spiralis* infection. Sequencing analysis showed that the ORF of TsDNase II-1 or TsDNase II-7 encode a protein of 347 and 348 amino acids, respectively, each protein contains a conserved domain.

Our results showed that TsDNase II-1 and TsDNase II-7 were successfully cloned and expressed in *E. coli*. After purification, rTsDNase II-1 and rTsDNase II-7 were immunogenic. Western blotting analysis showed that by using anti-rTsDNase II-1 and anti-rTsDNase II-7 sera, the two native DNase II enzymes were identified in the AW ES proteins and the crude extracts of various worm stages other than the ML. The qPCR analysis revealed that these two DNase II genes were transcribed throughout all the stages of the parasite’s lifecycle (ML, IIL, 3 and 6 days old AW, and NBL), with higher transcription levels in the AW stages and the lowest in the ML stage. The IIFT with anti-rTsDNase II-1 and anti-rTsDNase II-7 sera showed that the two native *Trichinella* DNase II were homogenously distributed along the external surface of *T. spiralis* AW and NBL stages, but not in the ML stages, and principally located at the cuticles and stichosome. The differential expression of TsDNase II-1 and TsDNase II-7 is likely due to the fact that the IIL, AW, and NBL stages lodge in the complicated enteral environment and penetrate the enteral epithelium and skeletal muscle. Whereas, the ML stage parasitizes within the nurse cells of the skeletal muscle, and the ML surrounding environment is stable; in addition to this, the larvae can survive for years without any major harm (Despommier, 1998). Another study showed that plancitoxin-1-like DNase II of *T. spiralis* was also located at the cuticle of the AW and NBL stages (Liao et al., 2014). Our results indicated that both the *Trichinella* DNase II investigated in this study are mainly located at the epicuticle; the presence of TsDNase II in the cuticle indicated that they are metabolically active, which can counteract the ETs consisting of DNA and proteases derived from the host’s macrophages and neutrophils during *T. spiralis* infection (Yousefi et al., 2008; Marin-Esteban et al., 2012).

Vaccination of mice with the two *Trichinella* DNase II enzymes induced the concurrent Th1/Th2 response and the obvious protection, as demonstrated by the significant reduction of adult and larva burdens. The protection may be due to fact that vaccination with *Trichinella* DNase II produced the TsDNase II-specific IgG, which neutralized the ability of DNase II to degrade the host’s DNA ETs and other proteases (Liu et al., 2008; Veerapathran et al., 2009; Li et al., 2015). Anti-TsDNase II IgG could also bind to the worm epicuticles and form the immune complex at the larval head, which may physically interrupt the larvae’s direct contact with IECs, therefore, block the worm invasion of intestinal mucosal columnar epithelium, and further lead to worm development (McVay et al., 1998, 2000; Song et al., 2018a,b). Our results demonstrated that *Trichinella* DNase II-specific antibodies evidently inhibited the parasite invasion of IECs. The *Schistosoma japonicum* DNase II contained a highly conserved catalytic domain, and rSjDNase II could degrade the genomic DNA and the elicited early humoral immune responses in mice (Hou et al., 2015). A similar result was also observed in a previous study on *Plasmodium* TatD-like DNase; the immunization of mice with *Plasmodium* TatD-like DNase elicited strong humoral responses and obvious protection against challenge with malarial parasite, and TatD-like DNase specific antibodies distinctly interrupted the exflagellation of male gametocytes, ookinete, and oocyst formation in the mosquito’s gut (Wang et al., 2018).

Specific anti-*Trichinella* antibodies could also kill *T. spiralis* NBL by an ADCC mode (Moskwa, 1999; Cui et al., 2015a). To evaluate cytotoxicity of anti-TsDNase II antibodies, we also carried out the *in vitro* ADCC experiment. The results demonstrated that TsDNase II specific antibodies took part in the NBL destruction. The PECs adhered to and damaged the NBL with the aid of TsDNase II specific antibodies, and the worm destruction was correlated with serum dilutions. It has been reported that the *P. berghei* TatD-like DNase can hydrolyze macrophage-derived ET structures and specific antibodies against *P. berghei* TatD-like DNase inhibiting *Plasmodium* proliferation in the host’s blood (Chang et al., 2016). The neutralization of the DNase enzyme activity by DNase specific antibodies may enhance the macrophage’s adhering and decomposing abilities on the parasites. The immune protection conferred by immunizing with rTsDNase II could be also because of the TsDNase II-specific antibodies neutralizing the TsDNase II enzymatic activity to degrade the ETs formed by the macrophages-derived DNA, which resulted in the killing and destruction of the parasite, impeded worm development, and therefore reduced the worm burden (Ortega-Pierres et al., 2015; Wang et al., 2018).

## Conclusion

The nucleases DNase II-1 and DNase II-7 are the proteins actively expressed at the adult and newborn larva stages during the lifecycle of the nematode *T. spiralis*. Immunization of mice with rTsDNase II-1 and rTsDNase II-7 generated a significant humoral immune response and protection after *T. spiralis* larval challenge. *Trichinella* DNase II-specific antibodies significantly inhibited the *in vitro* parasite invasion of enterocytes and participated in the killing of newborn larvae via an ADCC mode. The enzymes DNase II-1 and DNase II-7 might be related to worm development and as potential target molecules for the development of anti-*Trichinella* vaccine for both the inhibition of larval invasion and worm development. However, the oral vaccines of *Trichinella* DNase and the use of other safer adjuvants (such as Montanide ISA) need to be further investigated (Ortega-Pierres et al., 2015; Zhang et al., 2018).

## Author Contributions

ZW and JC designed this study. XQ, XY, YH, PJ, FY, JL, RL, and XZ conducted the experiments. XQ, ZW, and JC drafted and revised the manuscript. All authors read and approved the final version of this manuscript.

## Conflict of Interest Statement

The authors declare that the research was conducted in the absence of any commercial or financial relationships that could be construed as a potential conflict of interest.

## Footnotes

1 http://www.expasy.org/tools/protparam.html

## Supplementary Material

FIGURE S1 Expression and localization of TsDNase II-1 and TsDNase II-7 in the different stages of *T. spiralis* by IIFT. The frozen and intact worms of various stages were permeabilized by using 1% Triton X-100 for 5 min. The female adults were incised from the worm middle to release embryos on slides. The worms were incubated with 1:10 dilutions of anti-rTsDNase II-1 or anti-rTsDNase II-7 serum and dyed with anti-mouse IgG-cy3 conjugate (1:100). Subsequently, the slides were dyed with 4′, 6-diamidino-2-phenylindole (DAPI) at 37°C for 5 min. Immunostaining was observed on the surface of the worms of different stages (IIL, 3 and 6 days old AWs, embryos, and NBL). The muscle larvae incubated with *T. spiralis*-infected mouse serum or pre-immune serum was served as a positive or negative control.

TABLE S1 Inhibition of anti-rTsDNase II-1 and anti-rTsDNase II-7 sera on the *in vitro T. spiralis* IIL invasion of IECs.
