# Supplementary figures and images for: Characterization of Two Trichinella spiralis Adult-Specific DNase II and Their Capacity to Induce Protective Immunity
Source: Front Microbiol. 2018 Nov 5;9:2504. doi: 10.3389/fmicb.2018.02504 (PMC6230719; doi:10.3389/fmicb.2018.02504)

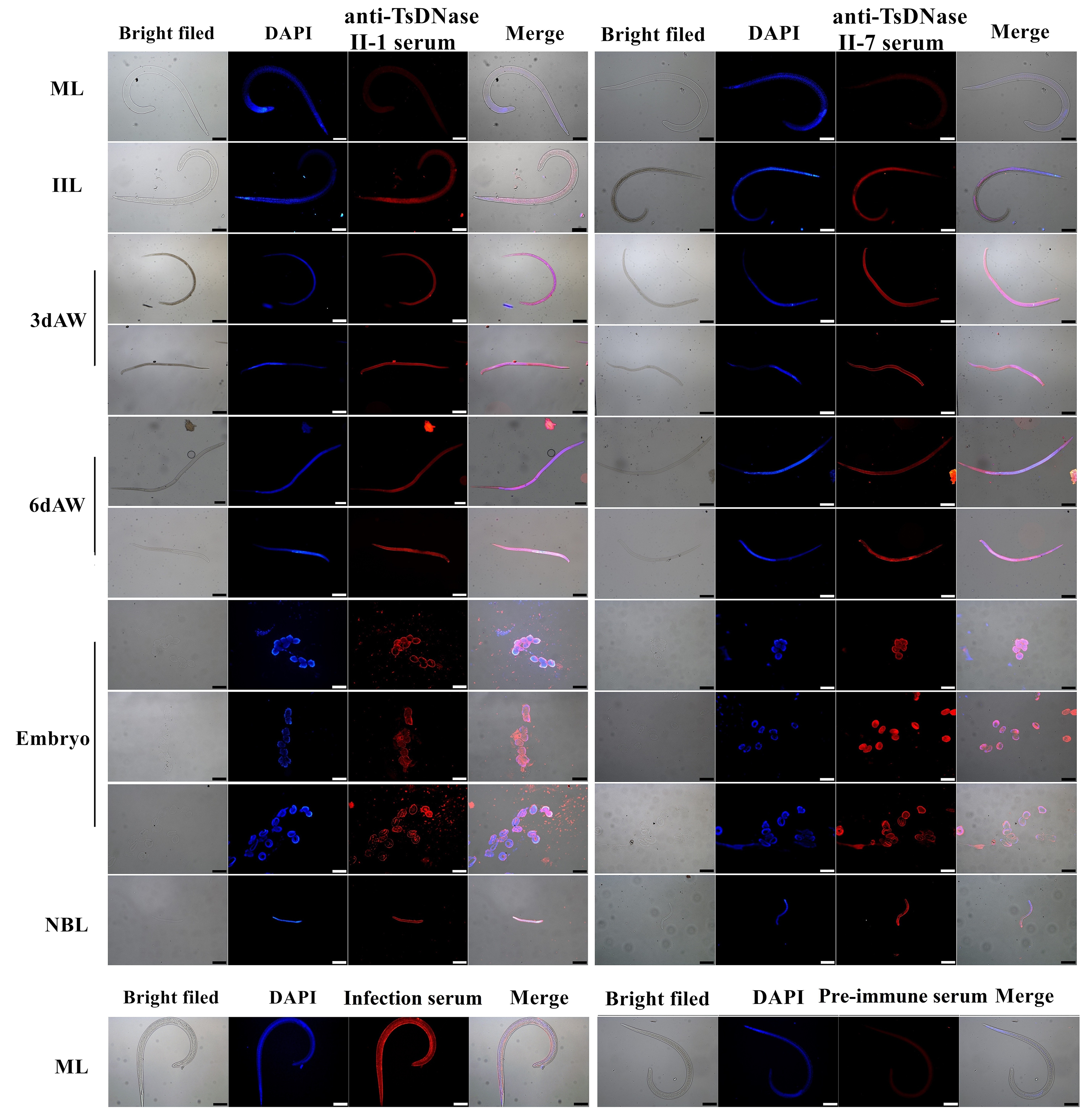

Supplement: FIGURE S1 — Expression and localization of TsDNase II-1 and TsDNase II-7 in the different stages of T. spiralis by IIFT. The frozen and intact worms of various stages were permeabilized by using 1% Triton X-100 for 5 min. The female adults were incised from the worm middle to release embryos on slides. The worms were incubated with 1:10 dilutions of anti-rTsDNase II-1 or anti-rTsDNase II-7 serum and dyed with anti-mouse IgG-cy3 conjugate (1:100). Subsequently, the slides were dyed with 4′, 6-diamidino-2-phenylindole (DAPI) at 37°C for 5 min. Immunostaining was observed on the surface of the worms of different stages (IIL, 3 and 6 days old AWs, embryos, and NBL). The muscle larvae incubated with T. spiralis-infected mouse serum or pre-immune serum was served as a positive or negative control. [file Image_1.TIF]
