# Supplementary material for: Characterization of Two Trichinella spiralis Adult-Specific DNase II and Their Capacity to Induce Protective Immunity
Source: Front Microbiol. 2018 Nov 5;9:2504. doi: 10.3389/fmicb.2018.02504 (PMC6230719; doi:10.3389/fmicb.2018.02504)
Supplement: TABLE S1 — Inhibition of anti-rTsDNase II-1 and anti-rTsDNase II-7 sera on the in vitro T. spiralis IIL invasion of IECs. [file Table_1.DOCX]

**Supplementary data- Table 1**

**Table 1** Inhibition of anti-rTsDNase II-1 and anti-rTsDNase II-7 serum on the *in vitro T. spiralis* IIL invasion of IECs

| **Type of serum** | **No. of larvae observed** | **No. of larvae invaded** | **Larval invasion rate (%)** |
| --- | --- | --- | --- |
| Anti-rTsDNase II-1 serum | 152 ± 2 | 79 ± 4 | 51.97 |
| Anti-rTsDNase II-7 serum | 153 ± 4 | 90 ± 4 | 58.82 |
| Infection serum | 152 ± 7 | 57 ± 5 | 37.50 |
| pre-immune serum | 159 ± 3 | 132 ± 5 | 83.02 |
| PBS | 159 ± 2 | 139 ± 8 | 87.42 |
